# Identification of a de novo* FOXP1* mutation and incidental discovery of inherited genetic variants contributing to a case of autism spectrum disorder and epilepsy

**DOI:** 10.1002/mgg3.751

**Published:** 2019-05-20

**Authors:** Kristy Jay, Amit Mitra, Taylor Harding, David Matthes, Brian Van Ness

**Affiliations:** ^1^ College of Biological Sciences, Department of Genetics, Cell Biology, and Development University of Minnesota‐Twin Cities Minneapolis Minnesota; ^2^ Department of Drug Discovery and Development, Harrison School of Pharmacy Auburn University Auburn Alabama; ^3^ College of Biological Sciences, Department of Biology, Teaching, and Learning University of Minnesota‐Twin Cities Minneapolis Minnesota

**Keywords:** *COMT*, *FOXP1*, *SLC6A4*, structural variants, whole genome/exome sequencing

## Abstract

**Background:**

Autism spectrum disorder is commonly co‐diagnosed intellectual disability, language disorder, anxiety, and epilepsy, however, symptom management is difficult due to the complex genetic nature of ASD.

**Methods:**

We present a next‐generation sequencing‐based case study with both de novo and inherited genetic variants and highlight the impact of structural variants on post‐translational regulation of protein expression. Since management of symptoms has classically been through pharmaceutical therapies, a pharmacogenomics screen was also utilized to determine possible drug/gene interactions.

**Results:**

A de novo variant was identified within the *FOXP1* 3′ untranslated regulatory region using exome sequencing. Additionally, inherited variants that likely contribute to the current and potential future traits were identified within the *COMT, SLC6A4*, *CYP2C19,* and *CYP2D6* genes.

**Conclusion:**

This study aims to elucidate how a collection of variant genotypes could potentially impact neural development resulting in a unique phenotype including ASD and epilepsy. Each gene's contribution to neural development is assessed, and the interplay of these genotypes is discussed. The results highlight the utility of exome sequencing in conjunction with pharmacogenomics screening when evaluating possible causes of and therapeutic treatments for ASD‐related symptoms.

## 1 INTRODUCTION

Autism spectrum disorder (ASD) is a complex developmental disorder defined by moderate to severe speech delay, deficits of motor coordination, and characteristic social and emotional behavior (Carr et al., 2010; Horn, 2011). ASD also commonly presents with comorbidities including epilepsy, intellectual disability (ID), and anxiety (Lee, Lee, & Kim, 2017). Difficulties in diagnosing and managing ASD include the large number of contributing genetic and environmental factors and broad spectrum of observable phenotypes (Xu, Cao, Zhang, & Cheadle, 2018). Recent studies have indicated that the heterogeneity of ASD can be understood through dysregulation of diverse cellular signaling mechanisms (Baudouin, 2014). Products of ASD‐associated genes may be members of critical developmental pathways, with deficiencies at varying genomic locations or developmental stages resulting in variable clinical presentation. While the genomic landscape of ASD has been increasingly well described in the post‐genomic era, the biological significance of many ASD‐associated variants has yet to be elucidated. There is an outstanding need to continue to accumulate genomic data toward the goal of understanding the complex context of ASD. While single case studies make complex variant predictions difficult, multiple genetic case studies that corroborate the significance of putative ASD‐associated developmental genes can confirm the significance of complex variant genotypes.

The two‐hit hypothesis of autism development involves compounding risks which can be of a genetic or environmental origin (Moussa, Srikrishnan, Blackwell, Dash, & Sibai, 2016). With variants in over 100 genes contributing to ASD (Lam, Yeung, & Law, 2017), a variant analysis pipeline of the genome or exome can suggest possible causative/contributing variants. Variants not observed in either parent are termed de novo mutations and occur at a rate of about 100 variants per child (Iossifov et al., 2013). Previous studies have linked chemical insults occurring during neural tube closure to ASD presentation (Rodier, Ingram, Tisdale, Nelson, & Romano, 1996), suggesting involvement of genes expressed during embryogenesis. Early central nervous system (CNS) patterning is determined by a number of transcription factors (TFs) including Forkhead Box P1 (*FOXP1*) (OMIM#605515). Deletions result in mild craniofacial abnormalities; a long narrow cranium, high and broad forehead, and short nose with broad bridge (Horn, 2011). FOXP1‐related ID syndrome is emerging as a unique disorder with affected individuals presenting with global developmental delay, specific craniofacial abnormalities, and heart or kidney malformations (Siper et al., 2017).

We present a familial case study involving an affected individual diagnosed with ASD, his parents and unaffected sister. From exome sequencing, we identified a potential de novo causative variant in the 3′ untranslated region (UTR) of *FOXP1* that we show likely impacts mRNA secondary structure and protein levels consistent with haploinsufficiency. The implications of the 3′ UTR variant are discussed in the context of other variants found in the same sequence repeat region of the gene, along with inherited variants that could contribute to the unique phenotype reported here. Additionally, inherited gene variations that could affect the phenotype and create adverse drug‐gene interactions were also identified using a pharmacogenomics screen as possible contributing factors.

## 2 METHODS

### 2.1 Ethical compliance

This study was approved by the University of Minnesota Ethics Committee. As an educational investigation, this study was considered exempt from requiring additional Internal Review Board approval. Samples from three family members were provided from a previous collection, with written consent to be used in this study.

### 2.2 Medical history

The patient (Patient 1), a 20‐year‐old male, was diagnosed with ASD by DSM‐IV criteria at age 2 years. He was born to nonconsanguineous parents, his father age 39, and his mother age 33 at the time of his birth. The patient's parents noted environmental risk factors including exposure to flood conditions in the home in early pregnancy and high levels of maternal stress throughout gestation. During pregnancy, his mother experienced significant bleeding at 36 weeks. He was born by cesarean section due to transverse lie, no other complications were noted during delivery. His sibling, a younger sister, and both parents are neurotypical. The proband, Patient 1, showed first signs of regression when he stopped tracking objects at 8 months. When he did not begin speaking at 12 months, he began speech and occupational therapy under the diagnosis of “autistic tendencies.” Patient 1 went on to speak short one‐syllable words and took his first steps at 13 months. Patient 1 was evaluated for hypertelorism, macrocephaly, prominent forehead, frontal hair upsweep, long narrow face, pronounced jaw, down‐slanting palpebral fissures, short nose, and frontal bridge. No dysmorphic craniofacial characteristics were reported. Patient 1 suffers from severe anxiety, hyperstimulation, and depression. He experiences difficulty transitioning and exhibits self‐injurious behavior when angry. These ASD‐related symptoms remain unresolved despite pharmaceutical treatment. Following formal diagnosis of “ASD and language disorder” at 2 years of age, Patient 1 was placed on multi‐vitamin supplementation along with the following: risperidone and aripiprazole without effect, methylphenidate, amphetamine/dextroamphetamine, dexamphetamine, catapres to treat hyperactivity, and gabapentin for anxiety. For anxiety a low dose clonazepam, and sertraline HCl have been tried, and he is currently taking citalopram. At the age of 17 years, following anesthesia for wisdom tooth removal, he began to experience seizure‐like activity. Frequency was initially weekly but improved with treatment of lamotrigine and lacosamide to every other month. Patient 1 recovered quickly from seizure‐like activity and did not experience loss of consciousness during episodes. Onset of seizure‐like activity was marked by slurring of speech and eyes rolling to the right. Several focal spikes in the front temporal lobe have been recorded on EEGs, and MRI results were normal. Parents report no family history of neurological disorders.

### 2.3 Exome sequencing

Genomic DNA was isolated from peripheral blood, obtained following informed consent, using QIAamp DNA Blood Maxi Kit (Qiagen Germantown, MD), quantified using Nanodrop‐8000 and stored at −20°C. Agilent XT libraries were captured using Agilent All Exon V5 + UTR kit. Libraries were pooled, and Exome sequencing was performed on a 125 bp paired‐end run in Illumina's HiSeq2500 next‐generation high‐throughput sequencing system using v4 chemistry. Variants identified through exome sequencing were submitted to the NCBI ClinVar database (https://www.ncbi.nlm.nih.gov/clinvar/).

### 2.4 Variant analysis pipeline

Raw next‐generation/Exome sequencing data were processed using an NGS variant analysis pipeline in Galaxy, a web‐based platform that provides tools essential to perform variant analysis. Prior to read alignment, data quality control (QC) was performed using pair‐end synchronization (pe‐sync) and FastQC tools in the Galaxy tools pane. Poor base call quality reads and adapter contamination were filtered using Adapter Removal (Lindgreen, 2012). Binary alignment mapping (.bam) files were then created by mapping raw Exome sequencing and RNAseq analysis reads to the hg19 human reference genome using Burrows Wheeler Aligner 0.5.9 (BWA) (Li & Durbin, 2009). Insert size distribution and coverage of on‐target fragments were determined using the Picard tool to assess the capture frequency. Ambiguously mapped and poor‐quality reads were removed using SAMtools. Reads were sorted, and mate‐pair information fixed using Paired read‐mate fixer tool within the Picard module that also removed duplicates. INDELs were realigned using Genome Analysis Toolkit (GATK) Indel realigner and then variants were called using GATK Unified Genotyper using a minimum phred‐scaled confidence threshold of 20 (Li et al., 2009; McKenna et al., 2010).

Since the proband is a Caucasian male with European ancestry, Western European (CEU) genomes in the 1,000 Genomes Project were used as controls to filter out common overlapping mutations (1000 Genomes Project Consortium et al., 2012). Inherited variants were analyzed and excluded as contributing factors through comparison of heterozygous/homozygous and missense variants of Patient 1 and his parents. The variant/vcf (variant calling format) file for Patient 1 was then examined for potential de novo causative mutations by comparing it with the variations present in both mother and father. The variants were annotated using the human reference database (GRCh38) and snpEFF to identify the most likely destructive variants (1000 Genomes Project Consortium et al., 2012; Cingolani et al., 2012). Candidate variants were further filtered based on the following criteria: variant novelty, genic or genomic location, data quality score (Qual), depth of coverage (DP), zygosity, phylogenetic conservation across species, percentage of reads with the variant, disease association, predicted splice site alterations, and predicted deleterious effects on protein and/or RNA processing (Worthey, 2013; Worthey et al., 2011).

### 2.5 Sanger sequence analysis

Buccal cells were obtained from Patient 1, both parents, and sister following informed consent. Genome extraction was completed on all samples using the protocol designed by (Mendoza et al., 2016). Primers for FOXP1 transcript 1 were designed: forward 5′‐AGATAGCCAGGAAGGCAGTG‐3′ and reverse 5′‐CATGTGGGAGGGAGAAACTC‐3′. Polymerase chain reaction (PCR) was completed using genomic template from each individual and gel extraction was utilized to isolate each sample using the manufacturers recommended protocol (Qiagen#28704). Samples were then submitted for Sanger sequencing at the University of Minnesota Genomics Center (UMGC). Forward and reverse reads were analyzed, and heterozygous base pairs (bps) were evaluated using Mutation Surveyor (SoftGenetics). Variants that could not be confirmed in the forward and reverse direction were excluded.

### 2.6 mRNA secondary structure

Secondary structure of the *FOXP1* messenger RNA (mRNA) (NM_032682.5:c.3413_3414del) (NM_032682.5:c.3413_3414dup) was evaluated using minimum free energy, and centroid algorithms accessed through the Vienna RNA package 2.0. Minimum Free energy thermodynamic analysis provided predicted secondary structures (RNAfold) to evaluate the effects of variation on secondary structure (Liu et al., 2014; Reamon‐Buettner, Cho, & Borlak, 2007).

### 2.7 Western blotting

Samples were obtained from Patient 1 and both parents following informed consent and peripheral blood mononuclear cells (PBMCs) were isolated using the Ficoll‐Paque gradient method. Nuclear extraction was completed following the manufacturer's recommended protocol (Abcam#113474), and the cytoplasmic fraction was used for western blot. To eliminate genomic contamination, each sample was treated with DNase 1 prior to electrophoresis. Western blots were completed using antibodies for *FOXP1* (D35D10, Cell Signaling Technology) and *PTEN* (D4.3, CST), with *β*‐actin (8H10D10, CST) used as a loading control, and a *FOXP1* overexpression lysate (LC40319, OriGene) used as a positive control. Protein concentration was determined using the Pierce BSA colorimetric assay (Thermo#23227). Samples (20 μg) were loaded on a 12% Bis Tris gel. Western blots were viewed using a Li‐Cor Odyssey imaging system (FC‐0470).

### 2.8 Pharmacogenomics testing

To assess possible side effects of pharmaceutical therapy enlisted in this case, a pharmacogenomics screen was completed. The RightMed comprehensive test (OneOme, Minneapolis, MN) was administered to determine possible unforeseen drug‐gene interactions that could result in an increase in the severity of symptoms or toxicity in the individual. Relevant variants identified through pharmacogenomics testing were submitted to the NCBI ClinVar database (https://www.ncbi.nlm.nih.gov/clinvar/).

## 3 RESULTS

### 3.1 Next‐generation sequencing of Patient 1 identified a de novo variant in *FOXP1* gene

Whole exome sequencing of Patient 1’s DNA was completed to identify possible ASD‐related genetic factors. To discount non‐causative variants, the exomes of both parents were also sequenced for comparative analysis. Exome sequencing called a total of 3,821,087 variants including 3,397,964 SNPs. A total of 2,717,840 of these variants followed the filtering criteria QUAL > 30. When the common overlapping mutations were filtered out using western European (CEU) genomes in the 1,000 Genomes Project data (1000 Genomes Project Consortium et al., 2012), 2,191,181 variants remained. Analysis of the remaining variants in Patient 1 was performed using the criteria: having a SnpEFF/SnpSift (Genetic variant annotation and effect prediction toolbox) and ClinVar (Archive of interpretations of clinically relevant variations) annotations, and with clinical significance and/or OMIM and 4,690 variants remained (Dolled‐Filhart, Lee, Ou‐yang, Haraksingh, & Lin, 2013; Landrum et al., 2014). Variants with at least one de novo mutant allele in the proband and with an autism‐related clinical annotation revealed rs143202281 (rs886058828) in the gene *FOXP1* (chr3: 71,004,983 GCA/G, GCA/CA (Qual = 46.73). Interestingly, clinical annotation identified FOXP1 as a candidate gene associated with the symptoms “Intellectual Disability with Language Impairment and Autistic Features” (Hamdan et al., 2010; LeFevre et al., 2013).

**Figure 1:**
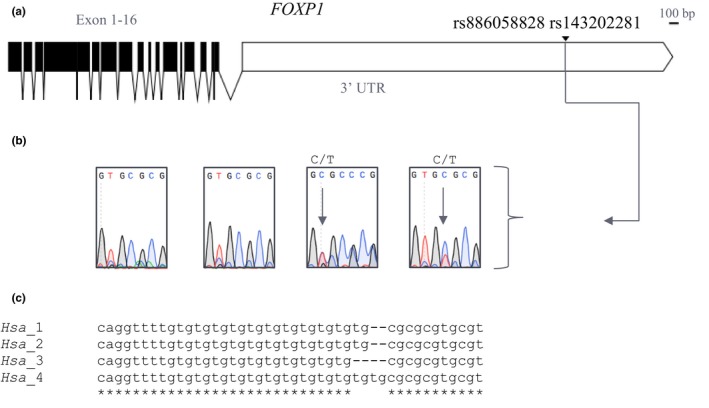
*FOXP1* Variant analysis (a) Exon map of *FOXP1*, including the 3′ UTR identifying the location of variants reported in Patient 1 (rs886058828) and his father (rs143202281). (b) Mother, sister, Patient 1, and father's Sanger sequencing results, respectively. Heterozygous insertion and deletion variants were reported in Patient 1 (NM_032682.5:c.3413_3414del) and his father (NM_032682.5:c.3413_3414dup). (c) Multiple sequence alignment of region of interest of the four family members, *Hsa* samples (1.) mother (2.) sister (3.) Patient 1 (4.) father

To validate our results, we performed PCR‐amplified Sanger sequencing using DNA from the proband, parents, and sister. Both mother and sister exhibited the common population sequence obtained from the NCBI ClinVar database, including 12 tandem (TG) repeats in the 3′ UTR of the *FOXP1* gene. A two‐base deletion following the TG repeat was reported in Patient 1, while curiously, a two‐base insertion was identified in his father (unaffected) at the same location, within the 3′ UTR of *FOXP1* (NM_032682.5:c.3413_3414del) (NM_032682.5:c.3413_3414dup). Figure 1 illustrates the heterozygous insertion and deletion variants reported in Patient 1 and his father, genomic location, and multiple sequence alignment of the four family members.

### 3.2 mRNA thermodynamic and centroid analysis revealed possible structural variants in *FOXP1* 3′ UTR associated with ASD

*FOXP1*’s 3′ UTR likely forms a complex secondary structure that regulates accessibility of RNA binding proteins and microRNA binding sites. We hypothesized that the 3′ UTR variant observed in Patient 1 may substantially alter *FOXP1* mRNA secondary structure and therefore impact *FOXP1* post‐transcriptional regulation. We queried the Vienna RNA secondary structure prediction software (Lorenz et al., 2011) with the *FOXP1* (NM_032682.5) variants identified above. Single base pairs were analyzed to identify regions that would self‐hybridize to form classical stem loop structures that could be targeted by RNA interacting proteins by predicting the minimum free energy (MFE) state of each sequence. Stability of the mRNA transcript is determined by hydrogen bonding between paired nucleotides (Gaspar, Moura, Santos, & Oliveira, 2013). A stable stem‐loop structure was identified in the variant region; and using MFE prediction, the most stable Δ*G* value for the local region in the mother and sister's transcripts was −33.8 kcal/mol. Notably, when the TG insertion (father) was analyzed, the Δ*G* value was indicated to be −34.0 kcal/mol. and the predicted structure was preserved. However, when the TG deletion (Patient 1) was analyzed, it was predicted that the secondary structure would be significantly altered, and the stem‐loop would be destabilized with a Δ*G* of −33.0 kcal/mol.

Analysis was then expanded to include a more global perspective of sequence variation on secondary structure, derived from other reported sequence *FOXP1* 3′ UTR variations. Centroid analysis was completed based on the Boltzmann‐weighted structure ensemble using the RNAfold webserver (Lorenz et al., 2011). mRNA structure of eight transcripts were completed; Patient 1, his father, and controls (mother and sister), and five additional variants submitted to the NCBI ClinVar database also associated with autism. These structures can be seen in Table 1, where the control structure is compared to the variant structures. It can be seen that in the variant observed in the father (NM_032682.5:c.3413_3414dup), the stem loop is preserved, while in all autism related cases it is lost or significantly altered.

**Table 1:** *FOXP1* mRNA structural analysis. A) *FOXP1* mRNA secondary structure predictions observed in mother and sister's (control) sequence. A stable stem‐loop structure can be seen with the TG tandem repeat identified in close‐up. (B) Patient 1 and his father's secondary structure predictions can be seen alongside additional variants from the NCBI ClinVar database

A
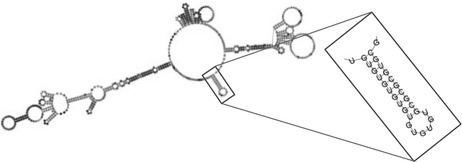

B					
Loc.	NCBI 1000 Genomes Browser	Variant	Associated Condition	Clinical Significance	mRNA Secondary structure
c.3403	rs886058829	del T	ID with language impairment and autistic features	Uncertain	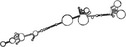
c.3407_3414	rs142202281	dup TGTGTGTG	ID with language impairment and autistic features	Uncertain	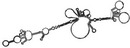
c.3411_3414	rs551672258	del TGTG	ID with language impairment and autistic features	Uncertain	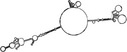
c.3413_3414	rs886058828	del TG (Patient 1)	ID with language impairment and autistic features	Uncertain	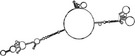
c.3413_3414	rs143202281	dup TG (Patient 1's father)	ID with language impairment and autistic features	Uncertain	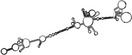
c.3415	rs886058827	delC ins TGTGTGTGTGT	ID with language impairment and autistic features	Uncertain	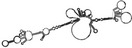
c.3419_3420	rs886058825	dup CG	ID with language impairment and autistic features	Uncertain	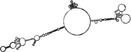

### 3.3 Western blotting confirms reduced FOXP1 protein expression in Patient 1

**Figure 2:**
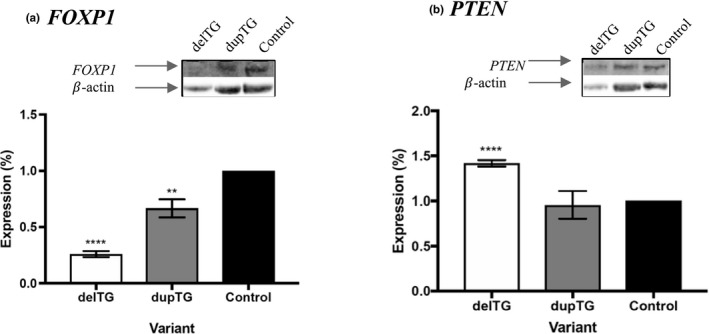
*FOXP1* Protein expression *FOXP1* and *PTEN* protein quantification between individuals. delTG indicates Patient 1, dupTG indicates his father, and the control indicates his mother. (a) *FOXP1* protein expression. (b) *PTEN* expression. In each case *β*‐actin was used as a loading control to normalize protein levels. Unpaired *t* test significance is denoted as *p *< 0.01(**) or *p *< 0.0001 (****) (*n* = 3, error = *SD*)

To explore *FOXP1* protein expression, western blot analysis was completed using PBMCs obtained from the Patient 1, and both parents. Of candidate interacting proteins, heterogeneous nuclear ribonuclear proteins (hnRNPs) would be associated with a decreased expression of *FOXP1*, while an overexpression could indicate decreased binding of microRNA‐486‐5p. Since microRNA‐486‐5p interacted with both FOXP1 and phosphatase and tensin homolog (*PTEN*) transcripts, we hypothesized that alteration in *FOXP1* interaction could lead to a microRNA‐486‐5p directed alteration of *PTEN* expression. Patient 1 exhibited a substantial (2.2‐fold) decrease in *FOXP1* protein, and elevated expression of *PTEN* (1.4‐fold) (Figure 2). An unpaired *t* test was carried out and *p*‐values were assigned for each of the variants for both *FOXP1* and *PTEN* expression. In the case of Patient 1 (NM_032682.5:c.3413_3414del), levels for both proteins were significantly different than the control with reported *p* < 0.0001. In his father's case (NM_032682.5:c.3413_3414dup), there was a much smaller difference in the level of *FOXP1* (1.4‐fold) decrease, and *PTEN* protein levels were not significantly different than the control levels observed in the mother.

### 3.4 Pharmacogenomics testing utilized to identify possible drug‐gene interactions

Pharmaceutical therapies are commonly used to manage behavioral symptoms associated with ASD. However, due to variants that influence metabolism, unforeseen drug‐gene interactions may occur including toxicity (Mitra, Dodge, Van Ness, Sokeye, & Van Ness, 2017). Since, medical interventions were being pursued for Patient 1, the RightMed comprehensive test (OneOme) was used to generate a personalized medication report summary, specifically targeting 23 genetic variants affecting at least 200 medications. This supplemented exome sequencing, since some of the analyses include copy number variants.

Among initial incidental findings, the gene and phenotype summary identified variants in the *CYP2C* Cluster (NC_000010.10:g.96405502G>A), the catechol‐O‐methyltransferase (*COMT*) gene (OMIM#116790) (NM_000754.3:c.472G>A), the coagulation factor II (*F2*) gene (NM_000506.4:c.97G>A), and the Solute Carrier Family 6 Member 4 (*SLC6A4*) gene (OMIM#182138) (NM_001045.5:c.‐1917_‐1875del43 and NM_001045.5:c.‐1936G>A). A summary of the variants reported in this case can be seen in Table 2, along with related drug impact and potential disorders. The full report from OneOme can be seen in Table S1.

**Table 2:** Patient 1 Drug Metabolism

Gene	Genotype	Phenotype summary	References
*CYP1A2* Cytochrome P450 Family 1 Subfamily A Member 2	*1A/*1F	Rapid metabolites may cause toxicity or side effects	RightMed Comprehensive test (OneOme)
*CYP2B6* Cytochrome P450 Family 2 Subfamily B Member 6	*1/*6	Decreased activity, active drugs converted to inactive metabolites may cause side effects or toxicity	RightMed Comprehensive test (OneOme)
*CYP2C19* Cytochrome P450 Family 2 Subfamily C Member 19	*1/*2	Decreased activity, active drugs converted to inactive metabolites may cause side effects or toxicity	RightMed Comprehensive test (OneOme)
*CYP2C* Cluster	rs12777823 GA	Altered warfarin clearance	RightMed Comprehensive test (OneOme)
*CYP2D6* Cytochrome P450 Family 2 Subfamily D Member 6	*1/*4	Decreased activity, active drugs converted to inactive metabolites may cause side effects or toxicity	RightMed Comprehensive test (OneOme)
*COMT* Catechol‐O‐Methyltransferase	rs4680 AA	Low activity in the catabolism of neurotransmitters dopamine, epinephrine, and norepinephrine	RightMed Comprehensive test (OneOme), NCBI Gene (www.ncbi.nlm.nih.gov/gene)
*F2* Coagulation factor II, thrombin	rs1799963 GA	Increased risk of thrombosis associated with prothrombin thrombophilia	RightMed Comprehensive test (OneOme)
*NUDT15* Nudix Hydrolase 15	rs116855232	Increased risk of leukopenia with thiopurine administration	RightMed Comprehensive test (OneOme)
*SLC6A4* Solute Carrier Family 6 Member 4	S/S (Sa/Sa)	Reduced expression of serotonin transporter localized in presynaptic neuronal membranes.	RightMed Comprehensive test (OneOme), OMIM (www.omim.org/entry/182138)
*UGT1A1* UDP Glucuronosyltransferase Family 1 Member A1	*1/*28	Decreased enzyme activity, increased risk for severe neutropenia with irinotecan and toxicity with nilotinib	RightMed Comprehensive test (OneOme)

Note

Gene/Phenotype summary of variants reported by the RightMed Comprehensive test (OneOme).

**Figure 3:**
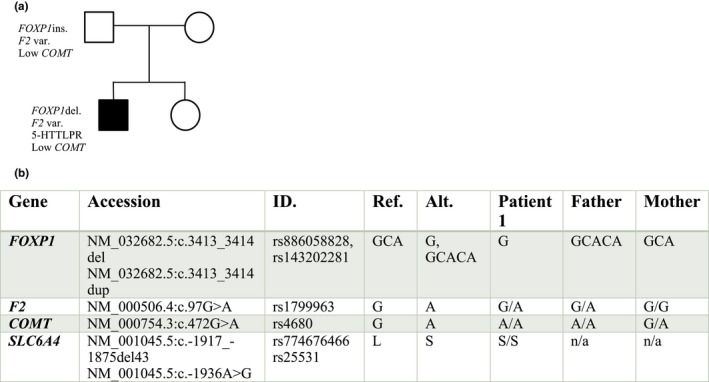
Contributing Genetic Factors Summary WES variant analysis pipeline results were identified and confirmed with the RightMed Comprehensive Test (OneOme). (a) Polygenic inheritance pattern pedigree. (b) Contributing gene summary including gene ID, chromosomal location, reference, and alternate SNP identifiers

Notably, some of these represent potentially important incidental findings. The *CYP2C* cluster and *NUDT15* (OMIM#615792) (NM_018283.3:c.415C>T) variants are strongly associated with therapies involving warfarin and thiopurines (Moriyama et al., 2016; Ndadza et al., 2018). These are not relevant to the case or management of behaviors but would be important entries in a permanent clinical document in case they may have future consideration. What was first considered incidental findings in the variants of *COMT* and *SLC6A4* genes, however, took on additional implications as discussed below. To stratify the extent of drug‐gene interactions, a comparison of current and previous medications aligned with metabolic enzyme pathway can be seen in Table 3. It was observed that citalopram and sertraline are metabolized by *CYP2C19* (OMIM#124020) (Uckun et al., 2015; Yuce‐Artun et al., 2016), and risperidone, aripiprazole, dextroamphetamine, dexamphetamine, and catapres are metabolized by *CYP2D6* (OMIM#124030) (Claessens et al., 2010; Dodsworth et al., 2018; Lisbeth et al., 2016; Miranda‐G. et al., 2007; Teh & Bertilsson, 2012). The RightMed comprehensive test reported Patient 1 as an intermediate phenotype for each of these genes, including decreased metabolic activity, stating that “drugs converted to active metabolites may have reduced efficacy. Active drugs converted to inactive metabolites may cause side effects or toxicity.” The Inheritance pattern for contributing genetic factors is included in Figure 3.

**Table 3:** Drug‐Gene interaction

Current treatment	Previous treatment	Function	Metabolism	Reference
Citalopram		SSRI antidepressant	*CYP2C19 *	Uckun et al., 2015
Lamitogrine		Anti‐epileptic (AED)	*UGT1A4*, *UGT2B7*, (*ABCB1* & *SLC22A1* transporter involvement)	Milosheska et al., 2016
Lacosamide		Anti‐epileptic (AED)	*CYP2C19* (no difference in *CYP2C19* poor metabolizers)	Kumar et al., 2017
	Risperidone	Antipsychotic	*CYD2D6 *	Dodsworth et al., 2018
	Aripiprazole	Antipsychotic	*CYP2D6 *	Lisbeth et al., 2016
	Methylphenidate	Stimulant (ADHD, ADD)	Carboxylesterase 1 (*CES1*)	Kaddurah‐Daouk et al., 2018
	Amphetamine & dextroamphetamine	Stimulant (ADHD)	*CYP2D6 *	Miranda‐G. et al., 2007
	Dexamphetamine	Stimulant (ADHD)	*CYP2D6 *	Teh & Bertilsson, 2012
	Catapres	Hypertension, ADHD, anxiety	*CYP2D6 *	Claessens et al., 2010
	Gabapentin	Anticonvulsant	Not metabolized	Radulovic et al., 1995
	Clonazepam	Anticonvulsant	*CYP3A5*, *CYP3A4 *	Tóth et al., 2016
	Sertraline	SSRI antidepressant	*CYP2B6*, *CYP2C19 *	Yuce‐Artun et al., 2016

Note

Pharmaceutical list of treatments for Patient 1 with metabolic pathways. Both current and previous treatments are included, and genes in which Patient 1 possesses a variant that affects metabolism are in bold.

## 4 DISCUSSION

Technological innovations in the post‐genomic era have radically enhanced our ability to identify potentially causative genomic alterations in complex and highly heterogeneous diseases and disorders. Identifying causative variants in ASD has proven particularly challenging and delineating the full genomic landscape of ASD necessitates in depth examination of countless additional individual cases. Through a targeted analysis pipeline, we identified variants within the 3′ UTR of the *FOXP1* gene at c.3413_3414 in both the proband and his unaffected father. Previously, variants within the functional domains of the *FOXP1* protein have been associated with autism (Carr et al., 2010; Hamdan et al., 2010; Horn, 2011); Pariani, Spencer, Graham, & Rimoin, 2009) and more recently, FOXP1 related ID syndrome (Meerschaut et al., 2018; Siper et al., 2017). For this reason, we identified FOXP1 as a possible causative variant in this case. The impact of these variants was then examined through mRNA thermodynamics analysis and quantified at the level of mature protein expression. The de novo variant in Patient 1 was identified using the WES variant analysis pipeline, and inherited variants were reported in possible contributing genes, supporting an increased risk of autism development with compounding risk factors. Indeed, this analysis demonstrates the potential for a combination of sequence variations having an impact on the developmental delays seen in this case and highlights the difficulty in assigning cause to a single gene‐trait.

*FOXP1* belongs to the Forkhead Box (FOX) superfamily of TFs, coding for proteins associated with neural development, metabolism, and immune function (Kumar, Batra, Kanthaje, Ghosh, & Chakraborti, 2017; LeFevre et al., 2013). Expression of genes that are transcriptionally regulated by *FOXP1* are dependent on maintaining a precise protein level. Although the exact mechanism of tissue‐specific regulation is yet undefined, *FOXP1* has been associated with motor neuron and striatum development and differentiation of dopamine neurons (Konstantoulas, Parmar, & Li, 2010; Li et al., 2015). FOXP2 was first implicated in the development of Language Disorder (LD), with haploinsufficiency linked to developmental verbal dyspraxia (DVD) (Feuk et al., 2006). *FOXP1* has been associated with more severe forms of ASD including ID (Araujo et al., 2017). Loss‐of‐function of *FOXP1* protein has been documented through reduced protein expression, reduced repressive activity, or improper alternate splicing (Meerschaut et al., 2018). Developmental titration of *FOXP1* levels can be regulated through numerous mechanisms. There is evidence that this is accomplished, in part, by post‐transcriptional regulation of *FOXP1* mRNA through critical cis regulatory elements (CREs) (Huelga et al., 2012; Jangi & Sharp, 2014; Liu et al., 2014) and microRNAs (miRNAs) in UTRs of the gene (Otaegi, Pollock, Hong, & Sun, 2011; Popovitchenko et al., 2016). Bioinformatic analysis revealed that the variants observed were near the binding sites of mRNA‐486‐5p (microRNA.org). The *FOXP1* pre‐mRNA is also subject to alternate splicing through hnRNPs (Huelga et al., 2012).

Comparison of NCBI ClinVar database variants in the local region identified additional cases of ASD presenting with expansion or contraction of the same TG tandem repeat sequence observed in this case. Structural prediction indicated that insertion and deletion variants could potentially alter the mRNA architecture. Diversity in the 3′ UTR of genes has been observed as one mechanism that mRNA provides variability in a tissue specific manner (Tushev et al., 2018). Further, genes such as *FOXP1*, with an unusually large 3′ UTR, could be subject to increased 3′ end processing to achieve this variability (Reamon‐Buettner et al., 2007). This study suggests that different 3′ UTR variants of *FOXP1* might share a common consequence on secondary structure and post‐translational regulation. Western blot analysis determined a twofold decrease in expression of the mature *FOXP1* protein in Patient 1, suggesting a potential role for *FOXP1* haploinsufficiency as a causative factor in this case (Figure 2).

*FOXP1* is expressed in the fetal telencephalon. *FOXP1* proteins interact with neural stem cells and promote differentiation and migration through repression of the Notch signaling pathway (Braccioli et al., 2017). *FOXP1* is also expressed in the developing striatal projection neurons and basal ganglia (Araujo et al., 2017; Tamura, Morikawa, Iwanishi, Hisaoka, & Senba, 2004). Reduction in the striatum and enlargement of the lateral ventricles has been observed in patients with *FOXP1* haploinsufficiency (Bacon et al., 2015; Pariani et al., 2009). *FOXP1* is necessary for proper development of motor circuits, and lack of FOXP1 in motor neuron progenitor populations may result in abnormal development of motor pools (Adams, Rousso, Umbach, & Novitch, 2015). Previous research suggests that determination of the preganglionic motor column and lateral motor column requires co‐repression of LIM homeobox (*LHX3/4*) genes and *FOXP1* (Mukaigasa et al., 2017). Since *FOXP1* is regulated in a tissue specific manner, studies including 3′ UTR variants could provide further insight into the pathophysiology of *FOXP1* deficiency within the CNS. Given that multiple affected individuals have presented with variants in this local region, the TG tandem repeat region observed could be a critical region for structurally relevant RNA‐protein interactions and have a pleiotropic downstream impact along a related developmental pathway.

While the de novo* FOXP1* variant was relevant based on previous associations with developmental disorders, the additional inherited variants we identified suggested additional considerations. The OneOme pharmacogenomic report provided additional potential insight into what was initially thought to be incidental findings (i.e., initially considered unrelated to the developmental delay in Patient 1). From that report we discovered Patient 1 carries an inherited dominant variant in the *F2* gene that is associated with a significantly increased risk of thrombosis (Lao, 2014). Reduced *COMT* expression, and the *SLC6A4* short transcript was also reported. Low fetal *COMT* expression, as observed in Patient 1, could create an added risk during vascular development and gestation and has been associated with preeclampsia and sudden infant death syndrome (SIDS) (Pertegal et al., 2016). This genotype has been associated with increased connectivity between the ventromedial prefrontal cortex and the amygdala (Klucken et al., 2015). *COMT* is responsible for catabolism of dopamine, norepinephrine, epinephrine, caffeine, and catechol estrogens (Sannino et al., 2015), and dopamine elevation has been observed in the autistic brain (Gardener, Spiegelman, & Buka, 2009). Dexamphetamine, as prescribed to Patient 1, functions by inducing the release of dopamine in the striatum, and dopamine and noradrenaline in the cortex (Schrantee et al., 2016). This incidental finding could influence the severity of symptoms observed in this case, and it is also possible that supplementation for low *COMT* could alleviate some of the anxiety Patient 1 is experiencing. Low *COMT* has been linked to anxiety and major depressive disorder. The neuroprotective and anti‐anxiety effects of magnesium are well established, and increased magnesium levels have been shown to neutralize surface charges in the CA1 neurons of the RETT mouse model (Balakrishnan & Mironov, 2018). Notably, low magnesium levels have also been shown to induce seizure‐like activity (Isaev et al., 2012).

The sodium dependent serotonin transporter (5‐HTT) is encoded by the *SLC6A4* gene and serotonin (5‐HT) homeostasis is involved in sleep cycle regulation, appetite, thermoregulation, pain perception, respiration, and bowel function (Margoob & Mushtaq, 2011). A 43 base pair insertion or deletion in the 5′ promoter region is called the 5‐HTT linked polymorphic region (5‐HTTLPR), denoted as either the short (S) or long (L) allele (Hooten, Hartman, Black, Laures, & Walker, 2013). The homozygous S allele as observed in Patient 1, would be expected to result in a reduction in translation of the serotonin transporter. Reduced expression is associated with decreased clearance of serotonin from the presynaptic cleft, and subsequent elevated extracellular fluid serotonin in the striatum, cortex, and hippocampal CA1 neurons (Murphy et al., 2008). Previous studies suggest that the *COMT* polymorphism, in conjunction with the *SLC6A4*‐HTTLPR S allele can result in a decrease in gray matter volume in the hippocampus, cerebellum, and striatum (LeFevre et al., 2013; Radua et al., 2013). During embryogenesis, 5‐HT neurons differentiate in the dorsal raphe nucleus (DRN) and migrate to the ventral forebrain through the medial forebrain bundle (Bonnin & Levitt, 2011). During the post‐natal refinement period, 5‐HT is expressed by the layer 5 & 6 pyramidal neurons (Andrade, 2011). The S allele of Slc6a4 has been shown to result in a reduction in mass of the prefrontal cortex, and abnormal connectivity, by increasing glutamatergic synapses on 5‐HT neurons (Soiza‐Reilly et al., 2018). Serotonin has also been shown to switch the attractive cue of netrin‐1 to repulsion in thalamic axon guidance (Bonnin & Levitt, 2011). Future studies could define the implications of elevated serotonin levels throughout development. Furthermore, maternal expression of the *SLC6A4* S allele has been associated with behavior development through stress induced hypermethylation that epigenetically alters the fetal transcriptome, downregulating critical genes involved in cortical development, and inducing an increased corticosteroid response to stress (Sjaarda et al., 2017).

Serotonin syndrome (SS) is caused by a progressive increase in serotonin levels in the central and peripheral nervous systems (Wang, Vashistha, Kaur, & Houchens, 2016). The Hunter Serotonin Toxicity Criteria (HSTC) includes the use of a serotonergic agent along with 1 of the 5 following: clonus, inducible clonus with agitation or diaphoresis, ocular clonus with agitation or diaphoresis, tremor, hypertonia with elevated temperature (above 38ºC) with ocular or inducible clonus (Volpi‐Abadie, Kaye, & Kaye, 2013). The serotonin transporter is inhibited by selective serotonin reuptake inhibitors (SSRIs) fluoxetine, sertraline, paroxetine, and citalopram (Rudnick, 2006), and serotonin release is stimulated by amphetamines (Vo, Nefsey, & Lin, 2015). Case reports indicate that the use of the cytochrome P450 2C19 (*CYP2C19*) inhibitor fluconazole can result in serotonin toxicity when taken with citalopram. Citalopram is a substrate for *CYP2C19* and inhibition of metabolism can result in toxicity (Levin, Cortes‐Ladino, Weiss, & Palomba, 2008). Patient 1 possesses a *CYP2C19* variant that reduces activity (Table 2), and is also taking citalopram, which could have adverse side effects. Inhibition of cytochrome P450 enzymes (*CYP2D6*) with SSRIs is another mechanism of SS, where an accumulation of serotonergic drugs (venlafaxine, methadone, tramadol, oxycodone, risperidone, dextromethorphan, and phentermine) leads to toxicity through decreased drug metabolism (Volpi‐Abadie et al., 2013). The RightMed comprehensive test also indicates Patient 1 possesses a variant in *CYP2D6* that would decrease enzymatic activity with side effects of toxicity (Table 2). These interactions suggest that pharmaceuticals affecting the same metabolic pathway could result in toxicity and may affect the seizure threshold.

These findings emphasize the importance of assessing both environmental and genetic risk factors and to evaluate possible drug‐gene interactions when enlisting pharmaceutical therapies. This study identified complex genetic variants that may represent possible causative drivers for ASD. Additionally, incidental findings through pharmacogenomic screening identified several variants that potentially have substantial implications for symptom management, emphasizing the importance of such screenings. Proper expression of TFs is of particular importance during pre‐natal and early post‐natal development, and deficits in neuronal migration have been implicated in autism, schizophrenia, and epilepsy development; however, the direct mechanism is yet unknown (Li et al., 2015). Here, we sought to identify putative genotypic variants that have the potential to influence neural circuitry, lower the seizure threshold and explain the unique neurological phenotype observed in this case. The biological significance of these variants is yet undefined and as additional patients are identified, may further suggest a pathogenic contribution to ASD development. Future studies should examine how neurological development is impacted by UTR variants in genes that encode proteins with putative dose‐dependent significance for ASD. Advancing our understanding of how an individuals’ genomic landscape both contributes to their specific neurological phenotype and determines their response to common therapies has the potential to dramatically improve informed treatment strategies and elevate the quality of life for individuals living with ASD.

## CONFLICT OF INTEREST

None to declare.
