# Supplementary material for: Identification of a de novo FOXP1 mutation and incidental discovery of inherited genetic variants contributing to a case of autism spectrum disorder and epilepsy
Source: Mol Genet Genomic Med. 2019 May 20;7(7):e00751. doi: 10.1002/mgg3.751 (PMC6625142; doi:10.1002/mgg3.751)
Supplement: Supplementary file 1 [file MGG3-7-e00751-s001.docx]

Supplemental Table 1.

OneOme Comprehensive Test Variant Summary for Patient 1 completed August 27, 2018

| Gene | 1000 Genomes Browser ID | Genomic Location |
| --- | --- | --- |
| CYP1A2 | rs2069514 | NG_008431.2:g.28338G>A |
|  | rs2069526 | NM_000761.4:c.-10+103T>G |
|  | rs12720461 | NM_000761.4:c.-10+113C>T |
|  | rs35694136 | NM_000761.4:c.-1635delT |
|  | rs762551 | NM_000761.4:c.-9-154C>A |
| CYP2B6 | rs3211371 | NM_000767.4:c.1459C>T |
|  | rs3745274 | NM_000767.4:c.516G>T |
|  | rs2279343 | NM_000767.4:c.785A>G |
|  | rs28399499 | NM_000767.4:c.983T>C |
| CYP2C9 | rs28371685 | NM_000771.3:c.1003C>T |
|  | rs1057910 | NM_000771.3:c.1075A>C |
|  | rs56165452 | NM_000771.3:c.1076T>C |
|  | rs28371686 | NM_000771.3:c.1080C>G |
|  | rs1057911 | NM_000771.3:c.1425A>T |
|  | rs1799853 | NM_000771.3:c.430C>T |
|  | rs7900194 | NM_000771.3:c.449G>A |
|  | rs9332131 | NM_000771.3:c.817delA |
| CYP2C19 | rs12248560 | NM_000769.2:c.-806C>T |
|  | rs28399504 | NM_000769.2:c.1A>G |
|  | rs4986893 | NM_000769.2:c.636G>A |
|  | rs6413438 | NM_000769.2:c.680C>T |
|  | rs4244285 | NM_000769.2:c.681G>A |
| CYP2C Cluster | rs12777823 | NC_000010.10:g.96405502G>A |
| CYP2D6 | rs1080985 | NM_000106.5:c.-1584C>G |
|  | rs1065852 | NM_000106.5:c.100C>T |
|  | rs59421388 | NM_000106.5:c.1012G>A |
|  | rs72549346 | NM_000106.5:c.1088_1089insGT |
|  | rs5030862 | NM_000106.5:c.124G>A |
|  | rs267608319 | NM_000106.5:c.1319G>A |
|  | rs774671100 | NM_000106.5:c.137_138insT |
|  | rs765776661 | NM_000106.5:c.1411_1412insTGCCCACTG |
|  | rs1135840 | NM_000106.5:c.1457G>C |
|  | rs201377835 | NM_000106.5:c.181-1G>C |
|  | rs769258 | NM_000106.5:c.31G>A |
|  | rs28371706 | NM_000106.5:c.320C>T |
|  |  |  |
|  | rs5030655 | NM_000106.5:c.454delT |
|  | rs5030865 | NM_000106.5:c.505G>[A,T] |
|  | rs3892097 | NM_000106.5:c.506-1G>A |
|  | rs72549353 | NM_000106.5:c.765_768delAACT |
|  | rs35742686 | NM_000106.5:c.775delA |
|  | rs5030656 | NM_000106.5:c.841_843delAAG |
|  | rs16947 | NM_000106.5:c.886C>T |
|  | rs5030867 | NM_000106.5:c.971A>C |
|  | rs79292917 | NM_000106.5:c.975G>A |
|  | rs28371725 | NM_000106.5:c.985+39G>A |
| CYP3A5 | rs2740574 | NM_017460.5:c.-392G>A |
|  | rs35599367 | NM_017460.5:c.522-191C>T |
| CYP4F2 | rs2108622 | NM_001082.4:c.1297G>A |
| COMT | rs4680 | NM_000754.3:c.472G>A |
| DYPD | rs55886062 | NM_000110.3:c.1679T>G |
|  | rs3918290 | NM_000110.3:c.1905+1G>A |
|  | rs67376798 | NM_000110.3:c.2846A>T |
| DRD2 | rs1799978 | NM_000795.3:c.-585A>G |
| F2 | rs1799963 | NM_000506.4:c.*97G>A |
| F5 | rs6025 | NM_000130.4:c.1601G>A |
| GRIK4 | rs1954787 | NM_001282470.2:c.83-10039T>C |
| HLA-A | HLA00097 | NM_002116 (interrogated at exon 2)  Negative |
| HLA-B | HLA00386 | NM_005514 (interrogated at exon 2 and intron 2) Negative |
|  | HLA00381 | NM_005514 (interrogated at exon 3) Negative |
|  | rs144012689 | NM_005514.7:c.1012+104A>T |
| HTR2C | rs7997012 | NM_000621.4:c.614-2211T>C |
| IFNL4 | rs12979860 | NM_001276254.2:c.151-152G>A |
| NUDT15 | rs116855232 | NM_018283.3:c.415C>T |
| OPRM1 | rs1799971 | NM_000914.4:c.118A>G |
| SLC6A4 | rs774676466 | NM_001045.5:c.-1917_-1875del43 |
|  | rs25531 | NM_001045.5:c.-1936A>G |
| SLC01B1 | rs4149015 | NM_006446.4:c.-910G>A |
|  | rs2306283 | NM_006446.4:c.388A>G |
|  | rs4149056 | NM_006446.4:c.521T>C |
| TPMT | rs1800462 | NM_000367.3:c.238G>C |
|  | rs1800460 | NM_000367.3:c.460G>A |
|  | rs1800584 | NM_000367.3:c.626-1G>A |
|  | rs1142345 | NM_000367.3:c.719A>G |
| UGT1A1 | rs4148323 | NM_001072.3:c.862-6536G>A |
|  | rs1976391 | NM_001072.3:c.862-9697A>G |
| VKORC1 | rs9923231 | NM_001311311.1:c.-1639G>A |
|  | rs7200749 | NM_024006.5:c.358C>T |

­Supplemental Table 1. Analytical results for Patient 1 from pharmacogenomics screen used to interpret possible drug-gene interactions (OneOme, Minneapolis, MN).
